# Melioidosis in Bihar, India: unearthing the first of many?

**DOI:** 10.1099/acmi.0.000260

**Published:** 2021-09-14

**Authors:** Isra Halim, Prathyusha Kokkayil, Ravi Kirti, Rajeev Nayan Priyadarshi, Asim Sarfraz, Binod Kumar Pati, Bhaskar Thakuria

**Affiliations:** ^1^​ Department of Microbiology, All India Institute of Medical Sciences (AIIMS), Patna, India; ^2^​ Department of General Medicine, All India Institute of Medical Sciences (AIIMS), Patna, India; ^3^​ Department of Radiodiagnosis, All India Institute of Medical Sciences (AIIMS), Patna, India

**Keywords:** *Burkholderia pseudomallei*, disseminated melioidosis, melioidosis

## Abstract

Melioidosis, a disease with protean clinical manifestations, is prevalent in many parts of India, with established endemic hotspots on the southern coast of the country. However, it is still underdiagnosed in many resource-poor regions of the country. We report what is, to the best of our knowledge, the first case of melioidosis diagnosed and treated in Bihar, an economically underdeveloped state in East India. The patient, a 52-year-old diabetic male, presented to the outpatient department with a fever of insidious onset along with pain and restriction of movement in the right shoulder joint and right knee joint, and swelling and tenderness of bilateral ankle joints. Radiological features were suggestive of multiple joint and organ abscesses. A diagnosis of disseminated septicaemic melioidosis was confirmed microbiologically. The patient improved clinically following aggressive treatment with meropenem and cotrimoxazole. The case highlights the need for increased clinical suspicion of melioidosis and adequate diagnostic facilities, as well as the need for early institution of appropriate empirical antibiotics in suspected cases of melioidosis in this region of the world.

## Case report

A 52-year-old painter presented to the outpatient department of a tertiary care teaching hospital with chief complaints of recurrent episodes of fever with chills throughout February 2021. He also complained of dull, aching, continuous, non-migratory pain in bilateral feet, the right knee, the right shoulder joint and the neck for the previous 20 days. This was accompanied by swelling of bilateral upper and lower limbs. The patient had a history of losing weight over the previous 4 years and uncontrolled diabetes mellitus for 8 years. He was also a heavy alcohol consumer (>5 drinks/day) and a chronic smoker. He was a resident of a rural district, Bhojpur, located in Bihar, East India, where he described himself as living in a kutcha house close to paddy fields. The patient belonged to a lower socioeconomic class corresponding to a score of 5 on a modified Kuppuswamy scale [1]. There was no history of a similar disease in the past, or among the other family members. The patient also gave no history of trauma, tuberculosis or other respiratory complaints, prolonged antibiotic use, or surgery in the past.

On presentation, the patient had a temperature of 37 °C, with a pulse rate of 78 beats min^−1^ and blood pressure of 98/72 mm of mercury. Examination revealed pallor and pitting oedema of the upper and lower limbs. On inspection, there was no erythema or discolouration of the skin over the limbs. Palpation of the knee and shoulder joint was acutely tender. The patient was unable to walk or abduct the right arm because of pain. Examination of other systems was unremarkable.

Random blood sugar on admission was 250 mg dl^−1^. Blood was collected for a complete haemogram, blood culture, renal function tests and viral serology. Haematological investigations revealed a total leukocyte count (TLC) of 17 000 µl^−1^, with 90.6 % neutrophils, and a platelet count of 38 000 µl^−1^. The patient had C-reactive protein (CRP) of 97.32 mg l^−1^ and glycosylated haemoglobin (HbA1C) of 13.53 %. Serum urea and creatinine were 53.5 and 0.98 mg dl^−1^, respectively, and the patient tested non-reactive for human immunodeficiency virus (HIV) and hepatitis B and C viruses. An ultrasound of the right lower limb revealed a 5.6 mm thrombus in the right saphenofemoral vein. A differential diagnosis of lower limb necrotizing fasciitis with deep vein thrombosis was made.

The patient was administered opioid analgesics to address excruciating joint pain. The antibiotics piperacillin/tazobactam, vancomycin and clindamycin were begun empirically for necrotizing fasciitis, in addition to insulin infusion and other supportive measures. However, within 48 h of admission, the patient became hypotensive and subsequently progressed to septic shock with acute kidney injury, requiring intensive care and inotropic support. Piperacillin/tazobactam was discontinued and the patient was started on meropenem 1 gm i.v. thrice daily.

**Fig. 1.**
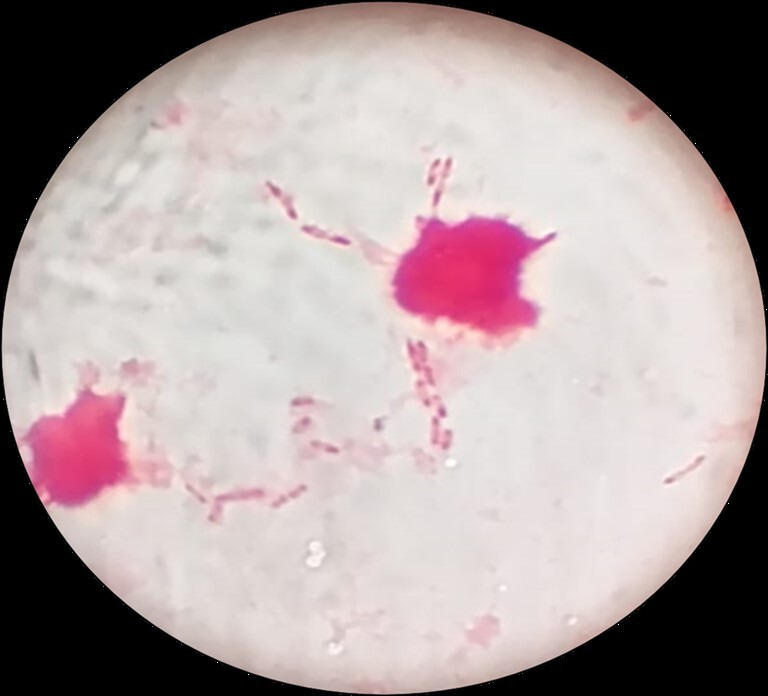
Gram stain of blood showing characteristic bipolar stained Gram-negative bacilli (safety-pin appearance).

**Fig. 2.**
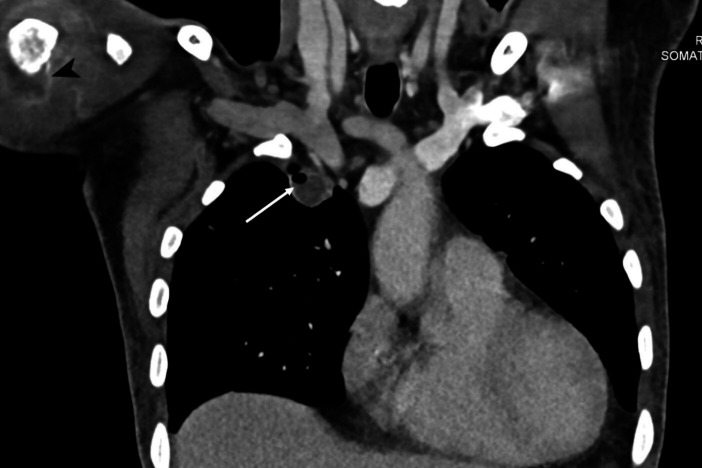
Computed tomography image (coronal view) showing a small lung abscess with air–fluid level (arrow) in the upper lobe of the right lung. Also, note the periarticular abscess around the right shoulder joint (arrowhead) that resulted from septic arthritis.

**Fig. 3.**
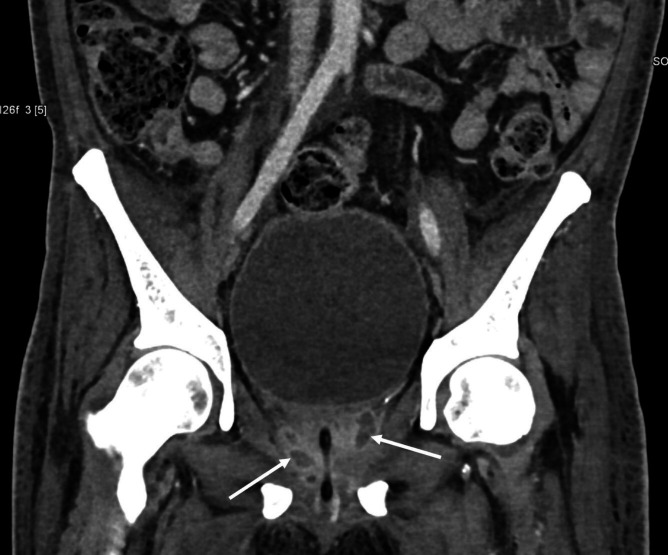
Computed tomography image (coronal view) showing multiple small prostatic abscesses (arrows) in the peripheral zone.

**Fig. 4.**
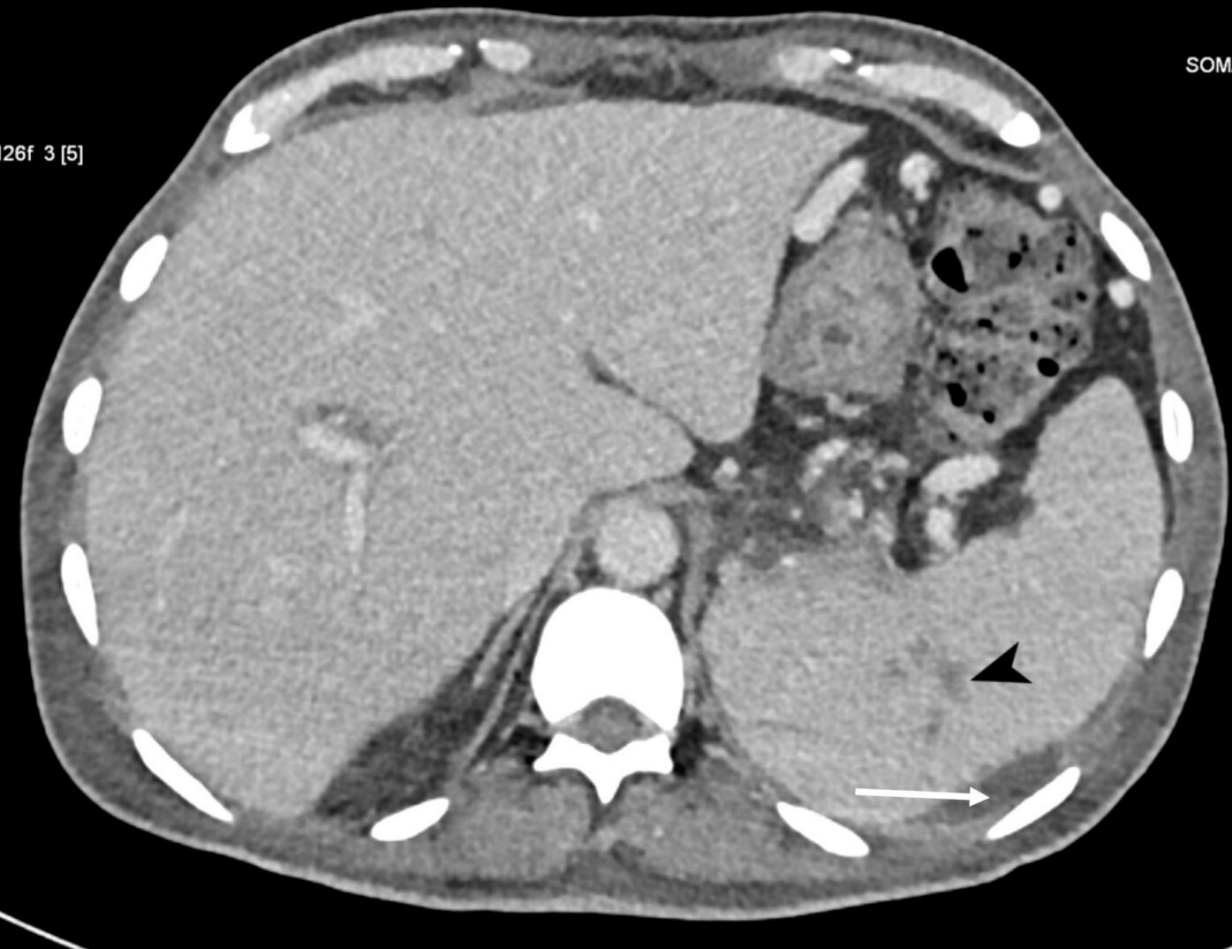
Computed tomography image (axial view) showing multiple small splenic abscesses (arrowhead) with minimal perisplenic fluid collection (arrow) that resulted from the abscess rupture.

Blood culture, collected soon after initial examination, flagged positive within 48 h of incubation. A Gram stain from the bottle showed bipolarly stained Gram-negative bacilli (Fig. 1). Within 48 h of incubation, growth on Blood and MacConkey agar showed medium-sized oxidase-positive colonies with dry margins and a metallic sheen. The organism was presumptively identified as *Burkholderia pseudomallei* by routine biochemical reactions and resistance to colistin, polymyxin B (300 units) and aminoglycosides, along with a sensitive zone size for amoxicillin/clavulanate. The identification was confirmed using the VITEK-II Compact system (bioMérieux, Marcy l'Etoile, France) with 98 % concordance. The isolate was uniformly susceptible to ceftazidime, meropenem, imipenem, doxycycline, and cotrimoxazole. The treating unit was promptly alerted about the organism. Antimicrobial therapy was revised by a multidisciplinary team consisting of physicians and microbiologists. The administration of injection meropenem (3 g/day) was continued, and injected vancomycin and clindamycin were stopped. Simultaneously, an urgent non-contrast computed tomography (CT) scan of the neck and thorax, and a contrast-enhanced CT of the abdomen, were planned to look for occult abscesses. Although the patient had no respiratory symptoms, radiological examination revealed multiple nodules in bilateral lungs with an abscess measuring 1.4×1.5×1.9 cm^3^ in the anterior segment of the right upper lobe, multiple soft tissue abscesses within muscles of the right shoulder joint, multiple prostatic abscesses and rim-enhancing splenic abscesses along with splenic vein thrombosis (Figs 2–4). Hence, the diagnosis was revised to disseminated septicaemic melioidosis with multiple deep organ abscesses and musculoskeletal involvement. The dose of injected meropenem was increased to 6 g/day, given the extent of the disease and the presence of deep organ abscesses. The patient showed gradual improvement over 1 week and was weaned off inotropes and intensive care. Urology and orthopaedic reference were sought for the prostatic abscesses, and possible septic arthritis of the knee joints, respectively. While a decision to allow spontaneous resolution of the prostatic abscess was taken, pig-tail drainage of the splenic abscess was considered. However, a day later, despite explaining the extent of the disease and the risk of fatality, the patient opted to be discharged. The patient was discharged on request following 14 days of intravenous meropenem. He was advised to continue treatment with intravenous meropenem for 4 more weeks, followed by an oral eradication therapy with cotrimoxazole for 3 months as recommended by the revised Darwin study consensus guidelines of 2020 [2], and to maintain adequate glycaemic control. The patient was followed up telephonically a month after diagnosis. He had been compliant with intensive intravenous therapy and showed symptomatic improvement.

## Discussion

Melioidosis is a well-known but underdiagnosed disease of the tropics and sub-tropics. The disease is endemic in parts of Australia, some Southeast Asian regions, and parts of the Indian subcontinent. The incidence of the disease has been rising steadily in India over the past decade, with many cases reported from the South Indian states of Karnataka and Tamil Nadu [3]. A community-based study from the southwestern coastal belt of India reported a seroprevalence of nearly 30 % among the adult population [4]. Furthermore, cases of imported melioidosis in tourists returning from this belt of the country have also been reported [5]. In contrast, the diagnosis of cases in the northern belt of India is infrequent, with only sporadic case reports. This disparity can be attributed to limited awareness about the disease, a lower index of clinical suspicion, and a lack of adequate laboratory support in this part of the country. There have been scattered case reports of melioidosis in patients from Bihar, an economically underdeveloped state in eastern India. However, these cases have been diagnosed at higher centres outside the state of Bihar (Table 1) [6–8]. A thorough literature search has not brought to light any published data on cases of melioidosis diagnosed and treated in Bihar. Our case, to the best of our knowledge, is the first autochthonous case of melioidosis to be diagnosed and managed in this region. Recent data highlight a dismally low prevalence of hospitalization for the treatment of infectious diseases in Bihar, compounding the problem of missing similar cases in the community [9].

**Table 1.** Review of melioidosis cases in patients belonging to Bihar, diagnosed at higher centres outside the state

Case	Place diagnosed	Age/sex	Risk factors	Clinical presentation	Treatment	Outcome
Mathew *et al.* 1999 [6]	Vellore	50/M	Diabetes	Splenic abscess	Ceftazidime Cotrimoxazole	Lost to follow-up
Barman *et al.* 2011 [7]	New Delhi	65/M	Diabetes	Disseminated septicaemic melioidosis	Imipenem+doxycycline Doxycycline+cotrimoxazole	Improved
Barman *et al.* 2013 [8]	New Delhi	65/M	Diabetes	Disseminated septicaemic melioidosis	Imipenem Doxycycline+cotrimoxazole	Improved
Barman *et al.* 2013 [8]	New Delhi	58/M	Diabetes	Disseminated septicaemic melioidosis	Imipenem Doxycycline+cotrimoxazole	Improved

The authors postulate that the state of Bihar could be a potential hotspot for the disease owing to conducive environmental factors such as soil, humidity and 1 million-odd hectares of total agricultural land, with a large population involved in farming. This hypothesis is supported by a recent modelling study that predicts the high environmental suitability for *B. pseudomallei* coupled with a background of suitable hosts in eastern India [10]. Over 75 % of Bihar’s population is employed in the agricultural sector [11]. Notably, Bihar is among the largest producers of rice in the country. The presence of *B. pseudomallei* in rice fields has frequently been described in the literature [12–15]. Rice farmers are known to be at high risk for melioidosis [16]. The mode of transmission of melioidosis includes inhalation, inoculation or ingestion of the bacteria [17]. Although our patient gave no history of farming, he would often walk barefoot in rice fields in his vicinity. The authors presume the patient could have been accidentally inoculated from such exposure. In addition to environmental determinants, host risk factors such as diabetes, alcoholism, chronic kidney disease and immunosuppression may also contribute to melioidosis in Bihar. Our patient had a history of prolonged uncontrolled diabetes and chronic hazardous alcohol intake. In a recent population-based cross-sectional survey, the prevalence of pre-diabetes and diabetes in Bihar was noted to be 10 and 4.3%, respectively [18]. The study also reported alcoholism in 18 % of males in the state. However, there are inadequate data on the true prevalence of several other risk factors, such as chronic kidney disease and immunosuppression.

The clinical presentation of melioidosis, the ‘mimicker of maladies’, includes fever, pneumonia, soft tissue or deep organ abscesses, septicaemia, musculoskeletal, osseous, genito-urinary and neurological complications [10]. Our case presented with disseminated melioidosis with multifocal abscess formation in muscles of the shoulder joint, spleen, lungs and prostate. Because of the diffuse involvement, non-specific symptoms, an uncommon presentation without obvious respiratory symptoms, and limited data on the occurrence of the disease in Bihar, melioidosis was not considered as a differential diagnosis on initial presentation.

The diagnosis of melioidosis remains a challenge in resource-constrained settings, reported cases representing only the tip of the iceberg. In addition to strong clinical suspicion, laboratories need to be well equipped to diagnose the disease. Culture from any specimen in a suspected case remains the gold standard for melioidosis. However, the growth of the organism in culture requires prolonged incubation and the need for selective media such as Ashdown agar for specimens from nonsterile sites. Specific biochemical reactions, and the typical resistance pattern of the organism to colistin, polymyxin B 300 and aminoglycoside antibiotics, along with susceptibility to amoxicillin/clavulanate, should prompt the clinical microbiologist to consider the diagnosis of *B. pseudomallei* in culture. Automated systems for identification, such as VITEK 2 Compact (bioMérieux, Marcy l'Etoile, France), BD Phoenix, or matrix-assisted laser desorption/ionization time-of-flight mass spectrometry (MALDI-TOF MS), are employed for identification, where available. In recent times, there has been a greater emphasis on rapid and accurate identification of the organism directly from clinical samples by employing molecular techniques real-time polymerase chain reaction (PCR) [19]. In the absence of advanced facilities for diagnosis, as in most resource-limited settings, strains of suspected cases of *B. pseudomallei* should be sent to reference centres for identification by automated identification platforms, PCR using targets such as type 3 secretion system-cluster 1 (T3SS-1) and sequencing. Multilocus sequence typing (MLST) would additionally help establish the genetic relatedness, if any, of strains from new geographical areas to Southeast Asian and Australasian sequence types, and could help recognize the association of different genotypes with varied presentations of the disease [20]. Rapid antigen tests, such as Active Melioidosis Detect™ (InBios, Seattle, WA, USA) lateral flow tests, may aid as a point of care test, especially in resource-poor settings with expected endemicity.

In the present case, the organism was isolated from the blood. Bacteraemia has been reported in 38–73 % of cases of melioidosis [10]. Imaging in cases of melioidosis is imperative to ascertain the multifocal dissemination of the disease. CT scans of the neck, thorax and abdomen in the present case revealed multiple abscesses in the lungs, spleen, soft tissues of the shoulder joint and prostate. Khiangte *et al*. report increased mortality in patients with imaging findings in the lungs [21]. They also report an increased association of splenic and musculoskeletal involvement in diabetic hosts, as was the case with our patient.

The management of melioidosis consists of an initial intensive phase and a prolonged eradication phase. *B. pseudomallei* from clinical isolates is seldom resistant to first-line antibiotics such as ceftazidime, doxycycline, amoxicillin/clavulanate, cotrimoxazole, imipenem and meropenem. According to the Revised Royal Darwin Hospital guide, the drug of choice for the intensive phase of melioidosis is intravenous ceftazidime or a carbapenem for an extended duration of up to 4 weeks in cases of disseminated disease with deep organ abscesses [2]. It would therefore be prudent to include ceftazidime or carbapenem as empirical therapy in patients from a reas endemic for melioidosis, and with a strong clinical or laboratory suspicion of melioidosis. The current case deteriorated initially, while on empirical antibiotic piperacillin/tazobactam. Clinical suspicion of melioidosis would have helped in the early initiation of meropenem at the appropriate dose. Gunasekaran *et al*. advocate the use of high-dose meropenem (2 g eighth hourly as 3 h infusion) in combination with therapeutic drug monitoring to manage septicaemic melioidosis successfully [22]. The eradication phase consists of the administration of oral antibiotics such as trimethoprim-sulfamethoxazole alone or in combination with doxycycline for a period of 3 to 6 months, depending on presentation. Despite a plan to continue intravenous antibiotics for 4 weeks, the patient requested discharge following 2 weeks of therapy. Unfortunately, the prolonged course of treatment with expensive antibiotics poses a treatment challenge in a state like Bihar, where a majority of patients are unable to afford therapy. Inappropriate intensive therapy and poor compliance with eradication therapy result in adverse clinical outcomes as well as disease relapse [23].

In a recently published review, Gassiep *et al.* speculate that India will lead the global melioidosis map by 2030, with the highest predicted number of annual melioidosis cases, synchronous with an alarming rise in the prevalence of diabetes from approximately 51 million to 87 million diabetics [19]. It is quite likely that we have been misdiagnosing or underdiagnosing numerous cases of melioidosis, limited by a lack of clinical suspicion as well as good diagnostic facilities. The present case highlights the importance of awareness of melioidosis as a cause of community-acquired septicaemia, local or systemic disease with multiorgan involvement requiring prompt diagnosis. Aggressive management is key to decrease relapse, morbidity and mortality due to the disease. Seroprevalence studies may help determine the extent of the disease in apparently naive population belts.

## Conclusion

This case report warns of the ominous presence of *B. pseudomallei* in the state of Bihar, India. A high index of suspicion, active case finding, collaborative teamwork between the physician and the clinical microbiologist, and adequate therapy are essential for diagnosis and favourable clinical outcomes in melioidosis.
